# Comparison of Repeated Fluid-Air Exchange and Passive Drainage for Removing Residual Emulsified Silicone Oil Droplets

**DOI:** 10.1155/2020/8184607

**Published:** 2020-07-04

**Authors:** Jian Yu, Yuan Zong, Ye Tan, Chunhui Jiang, Gezhi Xu

**Affiliations:** ^1^Department of Ophthalmology and Vision Science, Eye and ENT Hospital, Fudan University, Shanghai 200031, China; ^2^Key Laboratory of Myopia of State Health Ministry, and Key Laboratory of Visual Impairment and Restoration of Shanghai, Shanghai 200031, China; ^3^NHC Key Laboratory of Myopia (Fudan University), Key Laboratory of Myopia, Chinese Academy of Medical Sciences, Shanghai 200031, China; ^4^Gongli Hospital of Shanghai Pudong New Area, Shanghai 200135, China

## Abstract

**Purpose:**

To compare the efficacy of passive drainage (PD) and repeated fluid-air (FA) exchange for removing emulsified silicone oil (SO) droplets.

**Methods:**

Twenty rhegmatogenous retinal detachment (RRD) patients who underwent primary pars plana vitrectomy (PPV) with SO tamponade were randomly and evenly divided into two groups for PD and FA exchange. Once the bulk of SO was removed, the first 2 mL of the washout fluid was collected, and then, another 2 mL of the washout fluid was collected after PD or FA exchange. The size and number of SO droplets in the washout fluid were measured using a Coulter counter (Multisizer 3, Beckman Coulter, Indianapolis, IN, USA). The efficiencies of FA exchange and PD for removing emulsified SO were compared.

**Results:**

The number of SO droplets decreased significantly after FA exchange and PD. The decrease in the number of droplets was statistically significant for larger droplets (>4 *µ*m) with PD and for all droplet sizes with FA exchange. The decrease in the number of SO droplets of <4 *μ*m was significantly greater with FA exchange than with PD. However, at the end of the procedure, the overall number and size distribution of SO droplets were similar for both procedures.

**Conclusions:**

PD and FA exchange reduced the number of residual emulsified SO droplets after SO tamponade efficiently. FA exchange may have some advantages over PD in removing small droplets.

## 1. Introduction

Silicone oil (SO) was first introduced into vitreoretinal surgery by Cibis et al. [1] in 1962, and it has had a major impact on the surgical treatment of rhegmatogenous retinal detachment (RRD) [2, 3], diabetic retinopathy [4], and other vitreoretinal diseases [5–8]. However, several problems with SO have since been recognized, particularly the emulsification of SO, which was first reported by Haunt et al. in 1980 [9]. SO emulsification usually occurs a few months after surgery, but can happen quickly, sometimes within 1 week after surgery [10]. Other complications are also closely associated with SO emulsification including secondary glaucoma [11, 12] and keratopathy [13].

Consequently, many attempts have been made to reduce or prevent emulsification, including using highly viscous SO [14], incorporating very-high-molecular-weight polymers into SO [15], and reducing the duration of the SO tamponade [16]. Another approach that has been proposed involves removing emulsified SO droplets. Many methods have been developed, including passive drainage (PD) and fluid-air (FA) exchange [17, 18]. The efficiencies of these methods have been compared; Dabil et al. [17] reported that there were no differences in the efficiency of PD and FA exchange for removing emulsified SO droplets. More recently, Shiihara et al. [18] reported that FA exchange may increase the number of residual SO droplets. In both studies, the number of residual SO droplets was assessed in terms of objective symptoms or B-scan ultrasonography. However, these methods did not determine the number of residual SO droplets directly, and neither study assessed the number of droplets before PD and FA exchange. Therefore, the effectiveness of both methods must be clarified. Recently, Chan et al. [19] reported that SO droplets could be measured using a Coulter counter. In this study, we used a similar method to evaluate and compare the efficacy of PD and repeated FA exchange for removing emulsified SO droplets in patients who underwent primary pars plana vitrectomy (PPV) with SO tamponade.

## 2. Methods

### 2.1. Patients

Patients who underwent primary PPV with SO (Oxane 5700, Bausch & Lomb, USA) tamponade for RRD followed by uneventful SO removal at the Eye and ENT Hospital of Fudan University, Shanghai, China, were enrolled in this study. The same surgeon (Chunhui Jiang) performed PPV and SO removal in all patients. Best-corrected visual acuity, intraocular pressure (IOP), and fundus evaluation by slit-lamp microscopy with an indirect ophthalmoscopy lens (Maxfield 84 Diopter, Ocular Instruments, Bellevue, WA, USA) were assessed before SO removal and 3 months after SO removal. We collected information from medical records on patient characteristics, the time-course of primary PPV, duration of SO *in situ*, and axial length measured using an optical biometer (IOL Master, Carl Zeiss, Oberkochen, Germany) before SO removal. Patients were excluded if they had a history of intraocular disease other than cataract and RRD before primary PPV, were <18 years of age at primary PPV, had undergone cataract surgery within 6 months before the primary PPV, if retinal or other problems were found during SO removal that required air/SO tamponade, or if retinal redetachment occurred after SO removal. The study was approved by the Ethics Committee of the Eye and ENT Hospital of Fudan University and adhered to the Declaration of Helsinki. Informed consent was obtained from all patients. A total of 20 patients were enrolled and were randomly and evenly divided into the two procedural groups using a computer-generated list of random numbers.

### 2.2. Surgical Procedures

During SO removal, after the main bulk of SO was removed, the first 2 mL of washout fluid after was collected and FA exchange or PD were performed. FA exchange was performed by triple FA exchange as previously described [20], and then, air was replaced with the fluid. For PD, a 23-gauge back flute needle was inserted into the vitreous cavity, and the fluid was drained for 15 min at a pressure of 25 mmHg (Constellation 5000 Vision System, Alcon Laboratories, Inc., Fort Worth, TX, USA). After PD or FA exchange, another 2 mL of the washout fluid was collected, and the incision was closed.

### 2.3. Particle Collection and Measurement

As described by Chan et al. [19], we used a Coulter counter (Multisizer 3, Beckman Coulter, Indianapolis, IN, USA) to measure the size and number of droplets in the washout samples. This device simultaneously counts and measures the size of individual particles. For this study, we assessed the number of particles with diameters ranging from 1 to 12 *µ*m [19]. The values for each sample represent the mean of three consecutive measurements.

### 2.4. Statistical Analysis

The mean and standard deviations were calculated for the number of SO droplets and other clinical variables. The Mann–Whitney *U* test was used to compare clinical parameters and the number of SO droplets between the two procedures. The differences in the numbers of SO droplets before and after FA exchange or PD were determined using paired *t* tests, with the statistical significance set at *P* < 0.05. All analyses were performed using SPSS software version 20.0 (SPSS, Inc., Chicago, IL, USA).

## 3. Results

### 3.1. Patient Characteristics

All 20 patients were included in the final analysis, with 12 males and 8 females. The characteristics of the patients in the PD and FA exchange groups are shown in Table 1. Both groups were similar in terms of their age, sex distribution, axial length, duration of SO tamponade, and IOP before SO removal (all *P* < 0.05). The number of antiglaucoma eyedrops administered before and after SO removal was similar in both groups (all *P* < 0.05).

### 3.2. Efficacy of FA Exchange and PD

The total number of SO droplets and the distribution of droplets by diameter right after SO removal were similar in both groups (Table 2). The number of oil droplets decreased after both PD (Table 2) and FA exchange (Table 2). The number of droplets decreased significantly for larger droplets (>4 *µ*m in diameter), but not for smaller droplets (1–2 and 3–4 *µ*m) after PD (Table 2). In contrast, the decrease in the number of droplets following FA exchange was apparent for droplets of all diameters (Table 2). When we compared the changes in droplet distributions between the two procedures, we found that the decrease in the number (Table 3) and the percent decrease (Table 3) of SO droplets with diameters of <4 *μ*m were significantly greater for FA exchange than for PD, but the decreases in larger droplets were comparable for both groups. The numbers of residual SO droplets after PD or FA exchange were similar in both groups, regardless of the droplet size (Table 2).

## 4. Discussion

We compared the efficacy of PD and FA exchange for reducing the number of emulsified SO droplets in patients who underwent SO tamponade during PPV. We found that both procedures significantly reduced the number of residual SO droplets after SO tamponade, although FA exchange may show some advantages over PD in removing small droplets.

Emulsification of SO has long been an important focus of vitreoretinal surgeons, and many approaches have been proposed to estimate the number of emulsified SO droplets. Although most of the methods are either objective or semiquantifiable, Chan et al. [19] recently used a Coulter counter to count the number of SO droplets in the washout fluid collected after the removal of SO. This system is based on Coulter's principle [21] and can simultaneously count and measure the size and number of nonconducting particles suspended in a fluid. We used the same method to compare the efficiencies of PD and FA exchange for removing residual SO droplets. Both methods significantly reduced the number of SO droplets present in the vitreous cavity. Theoretically, both procedures could help to decrease the incidence of complications associated with emulsified SO droplets. Although further clinic trials with a larger number of patients and longer follow-up are still required, we think that both of these procedures could be used during SO removal. We also noticed that a large number of oil droplets were left in situ at the end of the procedures. This is consistent with the clinical finding that, even after these two procedures, the vitreous cavity may still contain many small, highly reflective particles that can be detected by B-scan ultrasonography [18]. Therefore, alternative methods still need to be developed to reduce or even eliminate the residual SO droplets.

Nevertheless, we identified some differences in the efficiencies of the methods. Although the number of residual droplets at the end of the procedure was similar in both groups, we found that the decrease in SO droplets of <4 *μ*m was significantly greater for FA exchange than for PD. This may imply that FA exchange is more efficient in removing the SO droplets from the eye. Although the reason for this is not clear, we propose an analogy of a clothing wash in which at the end of the rinse cycle, the clothes are spin-dried to provide a better cleaning result. Using FA exchange, the vitreous fluid is “spin-dried” three times.

Shiihara et al. [18] recently studied the effects of FA exchange on reducing residual SO, but unlike our study, their results were negative. They reported that compared with PD, FA exchange may increase the number of residual SO droplets. The reason for this was not completely clear, but the following explanation may be reasonable. First, there were differences in the methods used to evaluate residual SO droplets; they used a semiquantifiable method based on B-scan ultrasonography, whereas we used a Coulter counter to measure the number of droplets directly. Second, in their study, the decision to perform FA exchange and the amount of irrigated fluid were at the surgeon's discretion. In contrast, in our study, the patients were randomly assigned to the two groups, and we set a standard time for PD. These methodological differences may contribute to the differences in results between the two studies.

Our study was limited by its cross-sectional design and the limited number of patients in each group. Considering that newer methods of removing SO droplets have been developed, including the use of perfluorobutylpentane [22], further studies are required to evaluate the efficacy of these new methods. We should also acknowledge that, during the study period, no long-acting gas was available in China, so many of the patients were not very complicated cases, which may explain why there were no cases of retinal detachment during the follow-up.

## 5. Conclusions

In conclusion, our results indicate that PD and FA exchange are useful for removing residual emulsified SO droplets following SO removal. However, as a large number of droplets remained, newer methods with greater efficacy still need to be explored.

## Figures and Tables

**Table 1:** Patient characteristics.

Characteristic	FA exchange	PD	*P*
N	10	10	
Sex (M/F)	6/4	6/4	
Age (years)	49 ± 15	57 ± 9	0.162
AL (mm)	25.29 ± 2.07	26.07 ± 2.39	0.448
Preoperative IOP (mmHg)	16.4 ± 4.3	20.8 ± 8.4	0.184
Number of antiglaucoma eye drops before SO removal	1.5 ± 1.0	1.2 ± 0.9	0.487
Number of antiglaucoma eye drops number after SO removal	0.8 ± 0.9	0.2 ± 0.6	0.108
Duration of SO in situ (days)	483 ± 1095	174 ± 59	0.726

Values are expressed as the number of patients or mean ± standard deviation. FA, fluid-air; PD, passive drainage; M, male; F, female; AL, axial length; IOP, intraocular pressure; SO, silicone oil.

**Table 2:** The number of silicone oil droplets before and after fluid-air exchange or passive drainage.

	FA exchange			PD			*P* ^‡^	*P* ^§^
	Before	After	*P* ^†^	Before	After	*P* ^†^		
Number of droplets	1.17 × 10^6^ ± 0.83 × 10^6^	0.61 × 10^6^ ± 0.56 × 10^6^	**0.012**	1.58 × 10^6^ ± 2.64 × 10^6^	0.63 × 10^6^ ± 0.39 × 10^6^	0.305	1.000	0.280
Number of droplets with a diameter of								
1-<2 *µ*m	0.96 × 10^6^ ± 0.70 × 10^6^	0.51 × 10^6^ ± 0.48 × 10^6^	**0.016**	1.42 × 10^6^ ± 2.59 × 10^6^	0.51 × 10^6^ ± .35 × 10^6^	0.446	1.000	0.280
2-<3 *µ*m	1.34 × 10^5^ ± 0.94 × 10^5^	0.66 × 10^5^ ± 0.49 × 10^5^	**0.029**	1.03 × 10^5^ ± 0.41 × 10^5^	0.81 × 10^5^ ± 0.39 × 10^5^	**<0.001**	0.853	0.280
3-<4 *µ*m	4.18 × 10^4^ ± 3.17 × 10^4^	2.02 × 10^4^ ± 1.47 × 10^4^	**<0.001**	3.26 × 10^4^ ± 1.82 × 10^4^	2.36 × 10^4^ ± 1.23 × 10^4^	0.068	0.912	0.315
4-<5 *µ*m	1.52 × 10^4^ ± 0.93 × 10^4^	0.79 × 10^4^ ± 0.57 × 10^4^	**0.003**	1.13 × 10^4^ ± 0.52 × 10^4^	0.81 × 10^4^ ± 0.57 × 10^4^	**0.003**	0.631	1.000
5-<7 *µ*m	1.23 × 10^4^ ± 1.31 × 10^4^	0.62 × 10^4^ ± 0.53 × 10^4^	**<0.001**	0.81 × 10^4^ ± 0.36 × 10^4^	0.58 × 10^4^ ± 0.40 × 10^4^	**0.007**	0.853	0.853
7-<12 *µ*m	0.50 × 10^4^ ± 0.56 × 10^4^	0.24 × 10^4^ ± 0.14 × 10^4^	**0.004**	0.36 × 10^4^ ± .19 × 10^4^	0.20 × 10^4^ ± 0.17 × 10^4^	**0.012**	0.739	0.353

FA, fluid-air; PD, passive drainage. Values in bold are significant at *P* < 0.05. ^†^*P* value for within-group comparison of the change in number of droplets. ^‡^*P* value for between-group comparison of number of droplets before performing FA or PD. ^§^*P* value for between-group comparison of number of droplets after performing FA or PD.

**Table 3:** Decrease in the number and percent of silicone oil droplets following fluid-air exchange or passive drainage.

	FA exchange		PD		*P* ^†^	*P* ^‡^
	Change number	Change percent (%)	Change number	Change percent (%)		
Number of droplets	−0.56 × 10^6^ ± 0.55 × 10^6^	45.55 ± 20.62	−0.95 × 10^6^ ± 0.25 × 10^6^	28.36 ± 27.48	0.052	0.075
Number of droplets with a diameter of						
1-<2 *µ*m	−0.45 × 10^6^ ± 0.48 × 10^6^	44.54 ± 22.07	−0.91 × 10^6^ ± 0.25 × 10^6^	27.20 ± 29.20	**0.035**	0.063
2-<3 *µ*m	−0.68 × 10^5^ ± 0.70 × 10^5^	36.45 ± 9.31	−0.23 × 10^5^ ± 0.14 × 10^5^	26.90 ± 16.66	**0.035**	**0.015**
3-<4 *µ*m	−2.16 × 10^4^ ± 1.97 × 10^4^	46.54 ± 17.12	−0.90 × 10^4^ ± 1.47 × 10^4^	22.40 ± 21.11	**0.019**	**0.004**
4-<5 *µ*m	−0.73 × 10^4^ ± 0.56 × 10^4^	46.17 ± 21.10	−0.33 × 10^4^ ± 0.31 × 10^4^	31.57 ± 24.68	0.075	0.190
5-<7 *µ*m	−0.62 × 10^4^ ± 0.89 × 10^4^	44.46 ± 18.17	−0.23 × 10^4^ ± 0.25 × 10^4^	27.75 ± 30.11	0.123	0.190
7-<12 *µ*m	−0.26 × 10^4^ ± 0.45 × 10^4^	36.51 ± 22.87	−0.16 × 10^4^ ± 0.13 × 10^4^	44.21 ± 37.64	0.796	0.579

FA, fluid-air; PD, passive drainage. Values in bold are significant at *P* < 0.05. ^†^*P* value for between-group comparison of change in number of droplets. ^‡^*P* value for between-group comparison of percent change in number of droplets.

## Data Availability

The research data used to support the findings of this study are included within the article.
