# Erratum to “Comparison of Repeated Fluid-Air Exchange and Passive Drainage for Removing Residual Emulsified Silicone Oil Droplets”

**DOI:** 10.1155/2020/6495075

**Published:** 2020-11-02

**Authors:** Jian Yu, Yuan Zong, Chunhui Jiang, Ye Tan, Gezhi Xu

**Affiliations:** ^1^Department of Ophthalmology and Vision Science, Eye and ENT Hospital, Fudan University, Shanghai 200031, China; ^2^Key Laboratory of Myopia of State Health Ministry and Key Laboratory of Visual Impairment and Restoration of Shanghai, Shanghai 200031, China; ^3^NHC Key Laboratory of Myopia (Fudan University), Key Laboratory of Myopia, Chinese Academy of Medical Sciences, Shanghai 200031, China; ^4^Gongli Hospital of Shanghai Pudong New Area, Shanghai 200135, China

In the article titled “Comparison of Repeated Fluid-Air Exchange and Passive Drainage for Removing Residual Emulsified Silicone Oil Droplets” [1], there was an error in correspondence line. The correspondence line “Correspondence should be addressed to Chunhui Jiang; chhjiang70@163.com” is incorrect. The correct correspondence line should be “Correspondence should be addressed to Chunhui Jiang; chhjiang70@163.com and Ye Tan; tan.ye@live.cn.” This mistake was introduced during the production process, and Hindawi apologises for causing this error in the article.
